# The nutritional risk in oncology: a study of 1,453 cancer outpatients

**DOI:** 10.1007/s00520-012-1387-x

**Published:** 2012-02-07

**Authors:** Federico Bozzetti, Luigi Mariani, Salvatore Lo Vullo, The SCRINIO Working Group, Maria Luisa Amerio, Roberto Biffi, Riccardo Caccialanza, Giovanni Capuano, Isabel Correja, Luca Cozzaglio, Angelo Di Leo, Leonardo Di Cosmo, Concetta Finocchiaro, Cecilia Gavazzi, Antonello Giannoni, Patrizia Magnanini, Giovanni Mantovani, Manuela Pellegrini, Giuseppe M. Rovera, Lidia Rovera, Giancarlo Sandri, Marco Tinivella, Enrico Vigevani

**Affiliations:** 1 Faculty of Medicine, University of Milan, Milan, Italy; 2 Unit of Clinical Epidemiology and Trial Organization, Fondazione IRCCS Istituto Nazionale Tumori, Milan, Italy; 3 Ospedale Cardinal Massaia, Asti, Italy; 4 Istituto Europeo di Oncologia, Milan, Italy; 5 Fondazione IRCCS Policlinico San Matteo, Pavia, Italy; 6 Ospedale San Pietro Fatebenefratelli, Rome, Italy; 7 University Hospital, Belo Horizonte, Belo Horizonte, Brazil; 8 Istituto Clinico Humanitas, Rozzano, Italy; 9 Ospedale ASL 4 Prato, Prato, Italy; 10 Azienda Universitaria Senese “Le Scotte”, Siena, Italy; 11 Ospedale San Giovanni Battista, Turin, Italy; 12 Fondazione IRCCS Istituto Nazionale Tumori, Milan, Italy; 13 ASL 1-Ospedale Civile Carrara, Carrara, Massa Carrara Italy; 14 Ospedale S.Antonio USSL 16, Padua, Italy; 15 Policlinico Universitario Cagliari, Cagliari, Italy; 16 Presidio Ospedaliero Lucca, Lucca, Italy; 17 ASL10-Ospedale Civile Pinerolo, Pinerolo, Italy; 18 ASO Ordine Mauriziano Istituto di Ricerca e Cura del Cancro, Candiolo, Italy; 19 Ospedale S.Eugenio, Rome, Italy; 20 AOU San Luigi Gonzaga, Orbassano, Italy; 21 Ospedale S. Antonio Abate, Tolmezzo, Italy; 22 Fondazione IRCCS Istituto Nazionale Tumori, via G. Venezian 1, 20133 Milan, Italy

**Keywords:** Nutritional screening, Nutritional risk, Nutritional assessment, Nutritional status, Cancer outpatients

## Abstract

**Purpose:**

There is little information about the nutritional status of cancer outpatients because the practice of nutritional screening is rarely performed. This study aims to define the pattern of scores of nutritional risk in 1,453 outpatients and factors associated with a high nutrition risk score, to facilitate the identification of such patients by the oncologists.

**Methods:**

We prospectively screened the nutritional status of cancer outpatients according to the NRS-2002 score which combines indicators of malnutrition and of severity of the disease (1–3 points, respectively). A score ≥3 indicates “nutritional risk”. The association of the nutritional scores with some patient/tumour/therapy-related variables was investigated through univariable and multivariable linear regression models.

**Results:**

Thirty-two percent of outpatients were at nutritional risk. Primary tumour site, Eastern Cooperative Oncology Group score and presence of anorexia or fatigue were significantly associated with the nutrition risk score. Depending on the combination of these variables, it was possible to estimate different probabilities of nutritional risk.

**Conclusions:**

The frequency of a relevant nutritional risk was higher than expected considering the favourably selected population. The nutritional risk was associated with common clinical variables which are usually recorded in the charts and could easily alert the oncologist on the need of a further nutritional assessment or a nutritional support.

## Introduction

The European Society for Clinical Nutrition and Metabolism (ESPEN) defines nutrition risk [1] as “chances of a better or worse outcome from disease or surgery according to actual or potential nutritional and metabolic status” and nutritional screening [2] as a “rapid and simple process conducted by admitting staff or community healthcare teams”. The importance of the nutritional screening cannot be overlooked: the lack of routine screening procedure was shown to leave over half the patients who are nutritionally at risk unrecognised [3, 4] and one fourth without nutritional support or counselling despite the presence of an active contact of the patients with health care professionals [5]. Brown and Radke [6] in the USA and, more recently, Hulmann and Cunningham [7] in UK have emphasized the specific relevance of the nutritional screening also in the treatment of weight loss in cancer patients.

The Nutritional Risk Screening (NRS 2002) is a tool developed by Kondrup and an ESPEN working group in 2002 [2] with the assumption that the indications for nutrition support are severity of undernutrition and increase in nutrition requirements resulting from disease. It was designed to include measures of both current potential undernutrition and disease severity. It was validated against 128 controlled nutrition support trials to evaluate whether it was capable to distinguish those patients with a positive clinical outcome due to nutrition intervention from those that showed no benefit of nutrition support. Subsequently, a prospective, controlled trial with 212 hospitalised patients using the NRS 2002 [8] showed an increase in nutrition intake in patients who received nutrition intervention because they were defined at nutritional risk and a shorter length of hospital stay in those with complications (usually infections) who received a nutrition intervention comparing with those without nutritional support. Since its introduction in clinical practice, NRS 2002 (briefly NRS) was used to screen the nutritional risk of a mixed patients’ population and little attention was paid to the oncologic area and among them the outpatients.

However, since cancer patients represent the commonest segment of subject candidate to aggressive therapies both in the hospital and in ambulatory setting, they might present a state of malnutrition which can reduce the compliance to the oncologic therapies and can be also worsened by such treatments, this investigation focuses on screening the nutritional risk of a large population of cancer outpatients. The present study represents the final step of multicenter prospective protocol followed by the SCRINIO Working Group and aiming to define the pattern of scores of nutritional risk in a population of cancer outpatients, and to analyze the factors associated with a high nutrition risk score. In the perspective these data could help the clinician to identify patients at nutritional risk and hence to plan a nutritional intervention.

## Patients and methods

In 2003, during a scientific meeting in Milan, which involved both oncologists and nutritionists, it was clearly appreciated that there was a substantial discrepancy of view between these specialists as regard the impact that malnutrition might have on the outcome of the cancer patient and the potential role of the nutritional support. As a consequence, an open working group was constituted with the aim of steering a protocol to prospectively screen the nutritional status of the oncologic outpatients (hence the acronym SCRINIO, that is SCReenIng the Nutritional status In Oncology).

The endpoints of the study were: (1) to define prevalence and rate of malnutrition and of nutritional risk in cancer outpatients and the need for a nutritional intervention and (2) to investigate the association of some patient-related, tumour-related and therapy-related variables with the nutritional risk. The eligibility criteria included adult cancer outpatients presenting for diagnosis or therapy or follow-up to the oncologic units of different hospitals, university or scientific Institutions. Patients were excluded from the study if they were affected by endocrine diseases or they showed a severe impairment of vital organs’ function.

The protocol collected some demographic data (age and sex) of the patients, oncologic data [site of primary tumour, histology, stage (defined according to the UICC classification), Eastern Cooperative Oncology Group (ECOG) performance state, oncologic therapy] and nutritional data, namely the percentage of the weight loss at different interval times before and during the illness and the body mass index (BMI). Systemic and digestive symptoms as fatigue, anorexia, nausea/vomiting, early satiety, dysgeusia/dysosmia, odynophagia/dysphagia and diarrhoea/constipation were classified semiquantitatively through a four-point score (no, mild, moderate, severe).

Finally, the risk of complications related to malnutrition was assessed through the NRS. Briefly, if the patients at the initial screening have a BMI <20.5 Kg/m^2^ or they have lost weight in the last 3 months or they have a reduced dietary intake in the last week or they are severely ill, then they move to the final screening where a quantification of the previous parameters is completed and it summed with the severity of the disease. The final scoring ranges from 0 to 6, being 0 = no risk, 1–2 = low risk, 3–4 = medium risk and >5 = high risk (see Appendix 1). For age ≥70 years one additional score is added. A score ≥3 (we define it as “high NRS score”) is considered worth requiring a further deeper nutritional assessment for a potential nutritional intervention, whereas for a lower score a periodic nutritional surveillance is usually advised. This tool has been demonstrated to have a high predictive validity and a low interobserver variation (*k* = 0.76).

The study was implemented in 2004, included clinicians of 20 (mainly Italian) centres (see Acknowledgments) and closed to accrual on December 2008 after inclusion of 1,556 cancer patients. Of these, 103 lacked NRS information and were thus excluded from analyses. Each centre got the approval for the study and the informed consent form by its own local ethics committee. The central study database was held at the Fondazione IRCCS Istituto Nazionale Tumori of Milan where data collected on each patient were entered, checked for quality and completeness and elaborated. Details on the study protocol and preliminary results on the first 1,000 patients have been recently published [9], and database was previously utilized for two publications, one on weight loss and its association with digestive and systemic symptoms [10] and one on a definition and classification of cancer cachexia [11].

### Statistical methods

Descriptive analyses were based on standard statistics such as relative frequencies for categorical variables (gender, high NRS score, site of primary, UICC stage, ECOG performance status, therapy and symptoms degree) or otherwise with medians and interquartile ranges (NRS score, age). We considered anorexia in two different ways: “anorexia” as such, which simply means lack of appetite, and “anorexia syndrome”, also including symptoms interfering with food intake like early satiety, taste or smell alterations, nausea/vomiting and dysphagia/odynophagia [12]. The syndrome degree was defined as the maximum degree recorded for each of the contributing symptoms.

The patterns of association between NRS score and age, gender, site of primary, UICC stage, ECOG PS, therapy and symptoms were investigated by means of univariable and multivariable linear regression models. NRS score was alternatively treated as a continuous variable, or as categorical toward a classification threshold of 3 (NRS <3, NRS ≥3), denoting nutritional risk. Two-sided *P* values below 0.05 were considered significant. We used SAS™ and R software for computation.

## Results

Main series characteristics for the 1,453 patients with NRS score information, as shown in Table 1, were in general as expected for outpatients seen in the units of medical oncology, with a prevalence of advanced disease stage (III or IV according to the UICC classification, 80% of cases), good performance status (0 or 1 on the ECOG scale, 80% of cases) and some kind of oncologic treatment ongoing or completed (70% and 16% of cases, respectively). Symptoms were relatively common, but severe in only a minority of patients. As regards nutritional status, 32% of the patients were defined at nutritional risk that is 14% were with an NRS score >3, and another 18% were considered worthy of further nutritional assessment because of an NRS score = 3.

**Table 1:** Patient distribution according to demographic and disease characteristics

	*n*	%
Overall	1,453	
Gender		
Female	540	37.3
Male	908	62.7
N.R.	5	–
Age (years)^a^, median (IQ range)	64.0 (55–71)	
Site of primary tumour		
Oral cavity	116	8.0
Oesophagus	80	5.5
Stomach	206	14.2
Pancreas	90	6.2
Small bowel	33	2.3
Colon–rectum	518	35.7
Lung	217	15.0
Upper respiratory airways	84	5.8
Other	107	7.4
N.R.	2	–
UICC stage		
0	10	0.9
I	51	4.7
II	150	13.8
III	298	27.4
IV	578	53.2
N.R.	366	–
ECOG PS		
0	514	43.7
1	426	36.2
2	185	15.7
3	47	4.0
4	4	0.3
N.R.	277	–
Therapy		
Never treated	203	14.0
Past treated	234	16.1
Ongoing, one	838	57.7
Ongoing, two	164	11.3
Ongoing, three	14	1.0
NRS 2002 score		
0	368	25.3
1	336	23.1
2	287	19.8
3	259	17.8
4	138	9.5
5	53	3.7
6	12	0.8
Median (IQ range)	2 (0–3)	
Anorexia symptom		
No	683	47.2
Mild	362	25.0
Moderate	304	21.0
Severe	94	6.7
N.R.	7	–
Anorexia syndrome		
No	349	24.1
Mild	401	27.6
Moderate	477	32.9
Severe	224	15.4
N.R.	2	–
Fatigue		
No	455	31.4
Mild	536	37.0
Moderate	359	24.8
Severe	99	6.8
N.R.	4	–
Early satiety		
No	864	59.6
Mild	300	20.7
Moderate	221	15.3
Severe	64	4.4
N.R.	4	–

*N.R.* not reported, *IQ* interquartile, *UICC* International Union Against Cancer, *ECOG PS* Eastern Cooperative Oncology Group performance status

^a^Missing data in ten cases

Table 2 reports mean NRS score and percentage of patients with high NRS score according to distinct patients’ characteristics. In both cases, significant results were always achieved at the univariable and multivariable analyses, with tumour stage and therapy being the only exceptions. In particular, these two factors were no longer significant in the multivariable analysis of the percentage of patients with nutritional risk. Age (not shown) always failed to yield significant results.

**Table 2:** Mean NRS score and percentage of patients with nutritional risk (NRS score ≥3), according to main patients’ characteristics

	No. of pts.	Mean	*P*	Score ≥3	
				%	*P*
Factor					
Tumour site					
Oral cavity	116	1.5		28.5	
Oesophagus	80	3.1		62.5	
Stomach	206	2.3		43.7	
Pancreas	90	2.6	<0.0001^a^	54.3	<0.0001^a^
Small bowel	33	1.1	<0.0001^b^	6.1	0.0002^b^
Colon–rectum	518	1.5		24.3	
Lung	217	1.7		28.1	
Upper respiratory airways	84	1.4		28.6	
Other	107	1.4		25.2	
Tumour stage					
0	10	0.6		10.0	
I	51	1.4	<0.0001^a^	19.6	<0.0001^a^
II	150	1.5	0.0308^b^	23.3	0.1333^b^
III	298	1.8		33.2	
IV	578	1.7		29.8	
ECOG PS					
0	514	0.9		10.1	
1	426	2.0	<0.0001^a^	35.5	<0.0001^a^
2	185	3.3	<0.0001^b^	79.5	<0.0001^b^
3	47	4.0		91.5	
4	4	5.0		100.0	
Therapy					
Never treated	203	1.6		29.6	
Past treated	234	2.1	0.0017^a^	40.6	0.0052^a^
Ongoing, one	838	1.7	0.0742^b^	28.8	0.0669^b^
Ongoing, two	164	1.9		37.2	
Ongoing, three	14	2.1		35.7	
Anorexia					
No	683	1.0	<0.0001^a^	11.0	<0.0001^a^
Mild	362	2.0	<0.0001^b^	36.5	<0.0001^b^
Moderate	304	2.7		57.9	
Severe	97	3.5		79.4	
Anorexia syndrome					
No	349	0.8	<0.0001^a^	6.0	<0.0001^a,c^
Mild	401	1.5		22.0	
Moderate	477	2.2		41.5	
Severe	224	3.0		69.2	
Fatigue					
No	455	0.9	<0.0001^b^	11.4	<0.0001^a^
Mild	536	1.7	<0.0001^a^	23.5	<0.0001^b^
Moderate	359	2.6		58.2	
Severe	99	3.4		75.8	

^a^ *P* value from the univariable analysis

^b^ *P* value from the multivariable analysis

^c^Not included in the multivariable analysis

Table 3 shows the frequency estimated by the multivariable analysis according to tumour site, ECOG performance status and the presence of symptoms (anorexia and fatigue). As regards the remaining two variables, which failed to reach statistical significance in the multivariable analysis, we arbitrarily assumed tumour stage 3 and no therapy administration as reference categories to perform the calculation. Notably, the figures were obtained incorporating a statistically significant synergistic effect of anorexia and fatigue; namely, the joint presence of both symptoms was shown to have an impact on the frequency of patients at nutritional risk exceeding that expected by summation of their distinct contributions.

**Table 3:** Frequency of patients at nutritional risk (NRS score ≥3), estimated by the multivariable logistic model

Tumour site	Anorexia	Fatigue	ECOG performance status			
			0 (%)	1 (%)	2 (%)	3–4 (%)
Lower GI	Absent	Absent	7.9	26.0	56.5	73.7
		Present	15.2	42.2	73.0	85.4
	Present	Absent	10.7	32.8	64.4	79.6
		Present	37.0	70.5	89.9	95.0
Upper GI	Absent	Absent	15.4	42.6	73.3	85.6
		Present	27.5	60.7	85.1	92.5
	Present	Absent	20.2	50.8	79.3	89.2
		Present	55.4	83.5	94.9	97.6
Respiratory	Absent	Absent	9.6	30.0	61.4	77.4
		Present	18.0	47.2	76.8	87.7
	Present	Absent	12.8	37.4	68.9	82.7
		Present	41.9	74.5	91.6	95.9
Other	Absent	Absent	11.2	33.9	65.5	80.4
		Present	20.8	51.6	79.8	89.5
	Present	Absent	14.9	41.6	725	85.1
		Present	46.2	77.7	92.8	96.5

It is possible to observe that the frequency of patients with high NRS, 31.8% overall, was highly variable, ranging between a minimum of 7.9% to a maximum of 97.6%. To summarize, we found that conditions exceeding a 50% frequency threshold were ECOG performance status ≥2 (20.1% of the series), or ECOG performance status = 1 in the presence of both anorexia and fatigue (20.4% of the series); frequencies were generally much lower and below the 50% threshold in the remaining cases, with some detrimental effect of upper GI tumours compared to other tumour sites. For instance, an upper GI patient has an 85.6% probability of high NRS score if ECOG PS = 3–4; the probability is still high (83.5%) when ECOG PS = 1 but both symptoms are present, whereas the probability drops to 15.4% when ECOG PS = 0 and the patient is asymptomatic.

## Discussion

This study represents the first investigation using systematically the NRS 2002 to define the nutritional risk of cancer outpatients despite the guidelines by the American Society of Parenteral and Enteral Nutrition [13] that all patients should undergo nutritional screening dating back to 2002. These recommendations have been quite recently replicated in ad hoc ASPEN guidelines [14] and by the National Cancer Institute [15]. However, a recent survey in UK [16] showed that 80% of specialist oncological trainees expressed uncertainty or a lack of confidence in their ability to identify malnutrition and a similar study in US radiology oncologists [17] reported that only 9% of them used body weight plus other assessment tools.

Although several screening tools are available, there is no consensus among the experts upon the best way of screening the nutritional status of cancer patients and several of these tools, including the malnutrition screening tool [18–20], the malnutrition universal screening tool [21] and the patient-generated subjective global assessment [21–25], the subjective global assessment [21, 23, 26, 27] and the nutrition risk index [27, 28] are validated in oncology patients.

A large comparative study has shown that NRS 2002 has a better performance than the malnutrition universal screening tool and the nutrition risk index, compared to subjective global assessment [29]. Similarly, attempts to validate the malnutrition universal screening tool in a population with cancer showed that it was unsuitable for use because of low sensitivity and specificity [21, 30].

On the contrary, Sorensen et al. [31] have shown that the nutritional status determined by NRS 2002 maintained a significant independent association with complications even when adjusted for possible confounders as presence of cancer. We found that NRS 2002 is fully suitable for cancer population since many recognized prognostic factors (type of primary tumour, performance status, symptoms) parallel with the score of the NRS 2002.

The most striking finding of this study was that one third of our patients were considered with a high NRS, a percentage somewhat lower than the value (49%) recently reported by Isenring et al. [32] on a mixed population of 191 inpatients and outpatients. Our figure is intermediate between the values extrapolated for hospitalised cancer patients from large surveys of mixed pathologies populations, which range from 27% to 43% [20, 31, 33–35], while in advanced cancer patients enrolled in palliative home care services, the nutritional risk may rise till 68% [36]. Such a percentage of nutritionally at risk outpatients is especially remarkable and worrisome when considering that patients able to attend an ambulatory consultation or therapy should represent a favourably selected segment of the cancer population.

Since a score of 3 calls for a further more exhaustive nutritional assessment, it is noteworthy that a median value of 3 was observed in patients with cancer of the oesophagus and pancreas, in those with ECOG score ≥2 and in those with anorexia or fatigue of degree classified as moderate or severe. All of these factors achieved statistical significance also at the multivariable analysis (Table 2).

Even if oncologists do not feel comfortable, confident or adequately prepared to provide nutrition counselling, such a remarkable prevalence of outpatients with high nutritional risk should alert them to face actively with this issue for two reasons. First, the deleterious effects of malnutrition on compliance with oncologic therapies [37, 38] and response to treatment [39–45] are well recognized, and secondly, there is a growing experience that an early nutritional intervention when tumour burden is still limited is able to achieve a clinical benefit [46–48].

Finally, although undernutrition cannot entirely explain the progressive deterioration of the general status of the cancer patients, nevertheless, the correlation between NRS score and anorexia (which accounts for 76% of the NRS score ≥3) is in keeping with a potential role of nutrition support in decreasing the weight loss in the early stage of disease as shown in metabolic investigations [49, 50] and in RCTs [46–48]. Recently, Odelli et al. [51] reported that when applying a periodic nutrition assessment of all oesophageal cancer patient candidates to chemoradiation, they were able to identify patients at risk for malnutrition, to start early with nutritional support and to achieve a clinical benefit (less weight loss, greater radiotherapy completion, fewer and shorter unplanned hospital admission) as compared with a historical group of similar patients.

Furthermore Table 3 reports the probability for a patient to fall in a high NRS depending on the different combination of the site of primary tumour, presence or absence of some symptoms and values of the ECOG scale. It was not our intention to make a comparison with other screening methods, however it is worthy of note that these clinical variables together with some information on weight change, anorexia, performance status and gastrointestinal disturbances are commonly collected during the clinical oncologic examination and recorded in the chart. Moreover, body weight and height (and hence the BMI) are necessary to calculate the body area surface on which the doses of chemotherapeutics are calculated. Hence the presence of such variables can alert the clinician upon the potential onset of nutrition-related problems. In such a way, the oncologist is able to suspect any condition of nutritional risk and consequently depending on the severity of the situation, can provide the patient with some simple preventative suggestions (i.e. anti-anorectic/anti-cachectic agents or nutritional supplementations) or to defer him/her to a specialised nutrition support team.

There are some points of weakness in the study. Despite the high number of patients, this study does not involve all types of tumour, and patients with urogenital, bone and soft tissue malignancies are underrepresented. Hence our results cannot be generalised to all cancer population. It is true, however, that the variables we identified as significant (namely deterioration of the performance status, anorexia, fatigue) are expression of the metabolic component and the severity of the disease rather than a marker of a specific tumour and have a prognostic relevance in a variety of tumours.

Furthermore, oncologic treatments were disparate as regards type, administration and time interval from examination/interview of the patients, and we cannot exclude that some particularly aggressive therapies may adversely affect the nutritional risk. In such cases, however, the oncologist is usually aware of the impact of the oncologic treatment on the nutritional status and hence more than a screening procedure, a strict active surveillance of the occurrence of the potential nutritional complications is required.

In conclusion, this study shows that a large population of cancer outpatients, about one third, presents a high nutritional risk. Even though oncologists may be unfamiliar with nutritional tools or nutritional risk scores, nevertheless, the determination of some simple factors as site of primary tumour, the performance status and the presence of some symptoms (anorexia and fatigue) may alert them about a condition of nutritional deterioration of their patients and the potential use of a nutritional support.

## Appendix 1. Nutritional Risk Screening (NRS 2002)

**Table:**

Initial screening			
	Question	Yes	No
1	Is the BMI <20.5?		
2	Has the patient lost weight within the last 3 months?		
3	Has the patient had a reduced dietary intake in the last week?		
4	Is the patient severely ill (e.g. in intensive therapy)?		
Yes: If the answer is ‘yes’ to any question, the screening in Table 2 is performed.			
No: If the answer is ‘no’ to all questions, the patient is re-screened at weekly intervals. If the patient, e.g. is scheduled for a major operation, a preventive nutritional care plan is considered to avoid the associated risk status.			

**Table:**

Final screening			
Impaired nutritional status		Severity of disease (≈increase in requirements)	
Absent score 0	Normal nutritional status	Absent score 0	Normal nutritional requirements
Mild score 1	Wt loss 45% in 3 months or food intake below 50–75% of normal requirement in preceding week	Mild score 1	Hip fracture, chronic patients, in particular with acute complications: cirrhosis, COPD, chronic hemodialysis, diabetes, oncology
Moderate score 2	Wt loss 45% in 2 months or BMI 18.5–20.5 + impaired general condition or food intake 25–60% of normal requirement in preceding week	Moderate score 2	Major abdominal surgery, stroke, severe pneumonia, hematologic malignancy
Severe score 3	Wt loss 45% in 1 month (>15% in 3 months) or BMI <18.5 + impaired general condition or food intake 0–25% of normal requirement in preceding week	Severe score 3	Head injury, bone marrow transplantation, intensive care patients (APACHE >10)
Score	+	Score	=Total score
Age	If ≥70 years, add 1 to total score above	=Age-adjusted total score	
